# Microbial Diversity in Four Rhizocompartments (Bulk Soil, Rhizosphere, Rhizoplane, and Endosphere) of Four Winter Wheat Varieties at the Fully Emerged Flag Leaf Growth Stage

**DOI:** 10.1128/mra.00663-22

**Published:** 2022-10-06

**Authors:** Jabeen Ahmad, Athanasios Zervas, Lea Ellegaard-Jensen, Rosanna C. Hennessy, Ignazio Carbone, Vicki Cornish, Dorette Sophie Müller-Stöver, Amy Grunden, Carsten S. Jacobsen, Mette Haubjerg Nicolaisen

**Affiliations:** a Department of Plant and Microbial Biology, North Carolina State University, Raleigh, North Carolina, USA; b Department of Environmental Science, Aarhus University, Aarhus, Denmark; c Department of Plant and Environmental Science, University of Copenhagen, Copenhagen, Denmark; d Center for Integrated Fungal Research, Department of Entomology and Plant Pathology, North Carolina State University, Raleigh, North Carolina, USA; University of Maryland School of Medicine

## Abstract

Community composition and recruitment are important elements of plant-microbe interactions and may provide insights for plant development and resilience. The results of 16S rRNA amplicon sequencing from four rhizocompartments for four wheat cultivars grown under controlled conditions and sampled after flag leaf emergence are provided. Data demonstrate differences in microbial communities according to rhizocompartment.

## ANNOUNCEMENT

**FIG 1:**
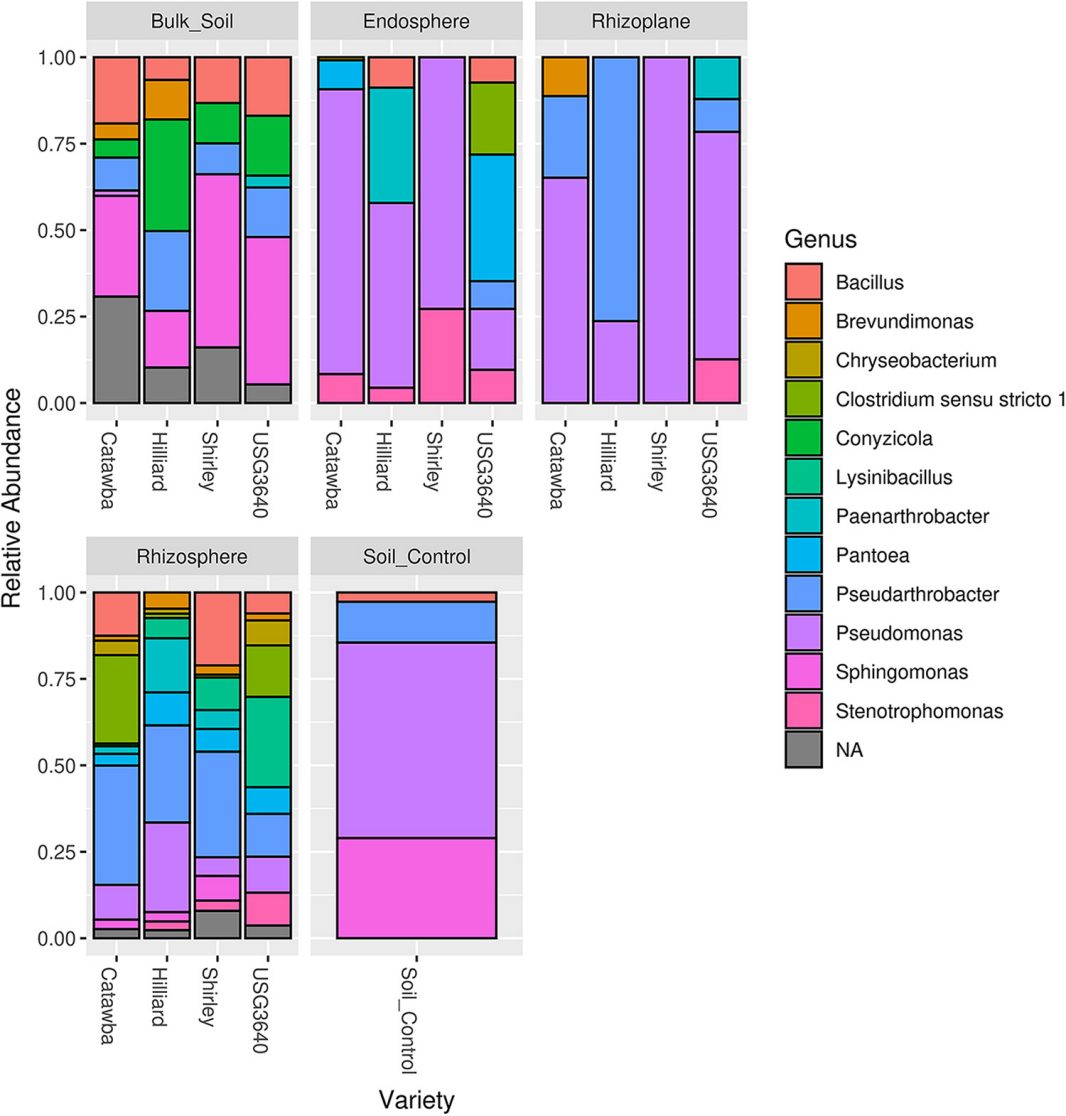
Microbial community compositions of the four rhizocompartments from the four wheat cultivars at the genus level. The figure shows relative abundances of the main genera for each variety in each rhizocompartment, as identified by 16S rRNA amplicon sequencing. NA, not assigned.

Wheat (Triticum aestivum L.) is an important crop for global food security and sustainability (1). Consumed for food, feed, and fuel, the demand for wheat has increased, requiring greater yields and implementation of strategies to reduce crop loss. Plant-microbe interactions in rhizocompartments may assist in wheat development and resilience to biotic and abiotic stress, improving production and mitigating losses (2). Research on wheat root microbiomes has intensified (3–5), but little is known about how microbial communities differ among rhizocompartments from different wheat cultivars. Here, we report on the diversity and structure of associated bacterial and archaeal communities based on amplicon sequencing of 16S rRNA gene libraries constructed from four rhizocompartments sampled from four wheat cultivars. The winter wheat varieties Hilliard, Shirley, Catawba, and USG-3640 were grown under controlled conditions in soil collected from the plough layer (0 to 25 cm) at the North Carolina State University Lower Coastal Plain Research Station (Kingston, NC, USA). Seeds of Hilliard, Shirley, and Catawba (sourced from Cunningham Field Station [Kinston, NC, USA]) and USG-3640 (provided by Mohamed Mergoum at the University of Georgia [Griffin, GA, USA]) were sown in field soil-filled D60H Deepots (https://stuewe.com/product/2-7-x-14-heavyweight-deepot-cell) with 100 individuals per cultivar (1 plant per pot). In addition, three pots contained only field soil to serve as controls. The varieties were sampled after the flag leaf fully emerged at Feekes stage 9.0 (6), at day 98 (Catawba), day 100 (Hilliard and USG-3640), and day 105 (Shirley). Using plant height as an indicator of plant performance, the five tallest and five shortest plants were sampled for each variety. For each plant, samples were collected for the four rhizocompartments (bulk soil, rhizosphere, rhizoplane, and endosphere) and the soil control. Samples were flash frozen, freeze-dried, and kept at 4°C until DNA extraction. DNA was extracted using the protocol from the NucleoBond RNA soil minikit (Macherey-Nagel, USA), and the concentration was measured with a Qubit 4 fluorometer. Amplicon libraries for Illumina sequencing were prepared via a two-step PCR (7) with the 341F/806R primer pair targeting the V3 and V4 hypervariable regions of the 16S rRNA gene (8) using the PCRBIO Ultra Mix (PCR Biosystems, UK). Samples were pooled equimolarly and sequenced on an Illumina MiSeq system using the MiSeq reagent kit v2 (500 cycles). Extensive details on the experimental setup, sampling process, laboratory work, and supplemental material are provided at https://github.com/biozervas/INTERACT_16S_USA_2021 or https://zenodo.org/badge/latestdoi/506132636. The numbers of reads varied from 8,104 to 387,736 across the 163 samples, with a length of 251 bp prior to trimming. Raw sequencing data were analyzed using DADA2 (version 1.8.0) (9) implemented in the DeCIFR toolkit (https://decifr.hpc.ncsu.edu) with default settings and the SILVA database (version 138) (10, 11) for taxonomic assignment. Sequencing data analysis using phyloseq (version 1.38.0) (12) showed that bacterial and archaeal community compositions differed among rhizocompartments and that microbial community compositions varied among varieties (Fig. 1). The generated amplicon data sets can be used to assess impacts of different rhizocompartments and varieties on the wheat root microbiome and to identify microbes associated with rhizocompartments and cultivars (Table 1).

**TABLE 1:** U.S. wheat sample varieties, compartments, and performance with corresponding SRA accession numbers

Variety	Root compartment	Performance^*a*^	SRA accession no. for runs
Soil control	Soil control	Soil control	SRR19128690, SRR19128689, SRR19128688
Hilliard	Rhizosphere	Best	SRR19128800, SRR19128799, SRR19128658, SRR19128647, SRR19128735
Hilliard	Rhizosphere	Worst	SRR19128724, SRR19128713, SRR19128702, SRR19128691, SRR19128680
Hilliard	Rhizoplane	Best	SRR19128687, SRR19128686, SRR19128685, SRR19128684, SRR19128683
Hilliard	Rhizoplane	Worst	SRR19128682, SRR19128681, SRR19128679, SRR19128678, SRR19128677
Hilliard	Bulk soil	Best	SRR19128734, SRR19128733, SRR19128732, SRR19128731, SRR19128730
Hilliard	Bulk soil	Worst	SRR19128729, SRR19128728, SRR19128727, SRR19128726, SRR19128725
Hilliard	Endosphere	Best	SRR19128772, SRR19128771, SRR19128770, SRR19128769, SRR19128768
Hilliard	Endosphere	Worst	SRR19128767, SRR19128766, SRR19128764, SRR19128763, SRR19128762
Shirley	Rhizosphere	Best	SRR19128798, SRR19128787, SRR19128776, SRR19128765, SRR19128754
Shirley	Rhizosphere	Worst	SRR19128743, SRR19128665, SRR19128661, SRR19128660, SRR19128659
Shirley	Rhizoplane	Best	SRR19128676, SRR19128675, SRR19128674, SRR19128673, SRR19128672
Shirley	Rhizoplane	Worst	SRR19128671, SRR19128670, SRR19128797, SRR19128796, SRR19128795
Shirley	Bulk soil	Best	SRR19128723, SRR19128722, SRR19128721, SRR19128720, SRR19128719
Shirley	Bulk soil	Worst	SRR19128718, SRR19128717, SRR19128716, SRR19128715, SRR19128714
Shirley	Endosphere	Best	SRR19128761, SRR19128760, SRR19128759, SRR19128758, SRR19128757
Shirley	Endosphere	Worst	SRR19128756, SRR19128755, SRR19128753, SRR19128752, SRR19128751
Catawba	Rhizosphere	Best	SRR19128657, SRR19128656, SRR19128655, SRR19128654, SRR19128653
Catawba	Rhizosphere	Worst	SRR19128652, SRR19128651, SRR19128650, SRR19128649, SRR19128648
Catawba	Rhizoplane	Best	SRR19128794, SRR19128793, SRR19128792, SRR19128791, SRR19128790
Catawba	Rhizoplane	Worst	SRR19128789, SRR19128788, SRR19128786, SRR19128785, SRR19128784
Catawba	Bulk soil	Best	SRR19128712, SRR19128711, SRR19128710, SRR19128709, SRR19128708
Catawba	Bulk soil	Worst	SRR19128707, SRR19128706, SRR19128705, SRR19128704, SRR19128703
Catawba	Endosphere	Best	SRR19128750, SRR19128749, SRR19128748, SRR19128747, SRR19128746
Catawba	Endosphere	Worst	SRR19128745, SRR19128744, SRR19128742, SRR19128741, SRR19128740
USG3640	Rhizosphere	Best	SRR19128646, SRR19128645, SRR19128644, SRR19128643, SRR19128642
USG3640	Rhizosphere	Worst	SRR19128641, SRR19128640, SRR19128639, SRR19128638, SRR19128736
USG3640	Rhizoplane	Best	SRR19128783, SRR19128782, SRR19128781, SRR19128780, SRR19128779
USG3640	Rhizoplane	Worst	SRR19128778, SRR19128777, SRR19128775, SRR19128774, SRR19128773
USG3640	Bulk soil	Best	SRR19128701, SRR19128700, SRR19128699, SRR19128698, SRR19128697
USG3640	Bulk soil	Worst	SRR19128696, SRR19128695, SRR19128694, SRR19128693, SRR19128692
USG3640	Endosphere	Best	SRR19128739, SRR19128738, SRR19128737, SRR19128669, SRR19128668
USG3640	Endosphere	Worst	SRR19128667, SRR19128666, SRR19128664, SRR19128663, SRR19128662

a Best or worst as determined by plant height.

### Data availability.

The 16S rRNA amplicon data set is available in GenBank and can be accessed in the SRA under the accession number PRJNA835863. Details on the experimental setup, sampling process, laboratory work, and supplemental material are available at Github (https://github.com/biozervas/INTERACT_16S_USA_2021) or Zenodo (https://zenodo.org/badge/latestdoi/506132636).
